# Prevalence of Occult Hepatitis C Virus Infection in Egyptian Patients with Lymphoma: A New Vision

**DOI:** 10.3390/diagnostics12041015

**Published:** 2022-04-17

**Authors:** Kholoud A. Elkashef, Wafaa A. Emam, Noha M. Mesbah, Dina M. Abo-Elmatty, Asmaa R. Abdel-Hamed

**Affiliations:** 1 Department of Biochemistry, Faculty of Pharmacy, Sinai University, El-Arish 45511, Egypt; 2 Department of Medical Biochemistry, Faculty of Medicine, Zagazig University, Zagazig 44519, Egypt; wafh22@yahoo.com; 3 Department of Biochemistry, Faculty of Pharmacy, Suez Canal University, Ismailia 41522, Egypt; noha_mesbah@pharm.suez.edu.eg (N.M.M.); dina_abouelmouti@pharm.suez.edu.eg (D.M.A.-E.); asmaa.ramdan@pharm.suez.edu.eg (A.R.A.-H.)

**Keywords:** lymphoma, occult HCV, peripheral blood mononuclear cells, RNA

## Abstract

Occult hepatitis C virus infection (OCI) is the absence of HCV RNA in serum and the presence of actively replicating HCV RNA in hepatocytes and peripheral blood mononuclear cells (PBMCs), as evidenced by the presence of antigenomic negative sense single-stranded RNA. This study aimed to determine the prevalence of OCI in Egyptian lymphoma patients and assess changes in biochemical parameters in patients with confirmed OCI. The current study was conducted on 100 apparently healthy subjects as control group and 100 patients with lymphoma as a case group. HCV RNA was extracted and detected in both plasma and PBMCs using qRT-PCR. Total protein, albumin, ALT, AST, and total and direct bilirubin were measured in serum. OCI was detected in 6% of the patient group. OCI patients had lower levels of total protein and serum albumin and higher ALT and AST compared with lymphoma patients without OCI. Our study revealed that six out of 100 patients with lymphoma disorders had occult HCV infection (6%). Therefore, the possibility of this infection should be considered in patients with lymphoma.

## 1. Introduction

Hepatitis C virus (HCV) is a leading cause of liver disease worldwide. It causes chronic viral hepatitis, which often leads to liver cirrhosis and hepatocellular carcinoma (HCC) [1]. HCV infection is Egypt’s most difficult public health problem, with the highest prevalence in the world [2]. HCV is a small, enveloped single-strand RNA virus with a positive sense that belongs to the *Flaviviridae* family, genus *Hepacivirus*. It infects human hepatocytes [3,4]. HCV is a hepatotropic and lymphotropic agent that causes a number of extrahepatic diseases, including B-cell lymphoproliferative disorders and liver injury, which can progress to cirrhosis and hepatocellular cancer [5].

Occult hepatitis C virus infection (OCI) was identified by Castillo et al. as a new type of chronic HCV infection [6]. Previous studies described occult HCV infection as the presence of HCV RNA in the liver and/or peripheral blood mononuclear cells (PBMCs) in the absence of circulating anti-HCV and HCV RNA [1]. Increased relapse rates in OCI patients support the theory that, after treatment, HCV can be sequestered in patients’ PBMCs or hepatocytes, effectively transferring the patient from chronic hepatitis C to OCI, where infection can then reactivate [7].

According to Petruzziello et al., the high prevalence of hepatitis C in Egypt (estimated anti-HCV prevalence of 14.7%) may be one reason for the high prevalence of OCI [8]. Although detecting OCI with HCV RNA in a liver biopsy specimen is the gold standard, testing for genomic and anti-genomic HCV-RNA strands in PBMCs has grown in popularity in recent years as an alternative technique that can be used when a liver biopsy specimen is not available [3].

Lymphomas are caused by chromosomal changes that cause uncontrollable proliferation of lymphoid cells [9]. Non-Hodgkin’s lymphoma (NHL), one of the two types of lymphoma, is the most common hematologic cancer in the world. Since the turn of the millennium, incidence rates have risen rapidly, with an annual percentage increase of about 3%, much faster than for most other cancers [10]. According to extensive epidemiologic studies and meta-analyses, HCV infection is a major factor in the development of some forms of B-cell NHL [11]

This study aimed to determine the prevalence of occult hepatitis C infection in Egyptian patients with lymphoma and determine alterations in biochemical parameters in lymphoma patients with confirmed OCI, with the goal of emphasizing the importance of HCV RNA testing in PBMC to identify the presence of OCI in lymphoma patients without anti-HCV or HCV RNA in serum as determined by routine methods.

## 2. Materials and Methods

### 2.1. Study Population

This study was conducted at Zagazig University’s Faculty of Medicine’s Medical Biochemistry and Oncology departments from April 2019 to March 2021. The study was conducted on 200 subjects divided into two groups: 100 newly diagnosed lymphoma patients divided into Hodgkin’s (25 patients) and non-Hodgkin’s (75 patients) and 100 apparently healthy volunteers. Lymphoma patients were selected from the Zagazig University hospital surgical oncology unit. Diagnosis was confirmed by biopsy and histopathological examination. Immunophenotyping was performed when differentiation was not observed by histopathological examination.

Healthy volunteers all participated in the Egyptian “100 million health” campaign, an extensive screening initiative that screened 57 million citizens for anti-HCV antibodies.

A detailed history including name, age, sex, occupation, residence, history of previous drug administration, and other liver problems was documented for all participants.

None of the study participants had a history of hepatocellular carcinoma, liver cirrhosis, or previous HCV infection.

Selection criteria for patients were as follows: patients with confirmed lymphoma, negative for anti-HCV antibodies, hepatitis B core antibody (anti-HBC), HBV DNA, anti-human immunodeficiency virus (HIV) antibodies, Epstein–Barr virus (EBV), and cytomegalovirus (CMV) (obtained from patient files as a routine workup for newly diagnosed lymphoma patients). Control group inclusion criteria were apparently healthy individuals, negative for serum HCV Ab. Exclusion criteria were positive results for HBVsAg, HIV, Epstein–Barr virus (EBV), or cytomegalovirus (CMV).

### 2.2. Sampling

Seven milliliters of peripheral blood were drawn from each participant and divided into two portions; 2 mL were collected into plain tubes for serum separation and measuring biochemical parameters including alanine aminotransferase (ALT), aspartate aminotransferase (AST), total bilirubin, direct bilirubin, total protein, and albumin, while 5 mL were collected into heparinized tubes for the separation of peripheral blood monocytes and plasma for HCV RNA extraction and detection.

#### 2.2.1. Serum Separation

Whole blood was left to clot for 30 min at room temperature and then centrifuged at 3000× *g* for 30 min at 4 °C. Supernatant was collected into sterile tubes and centrifuged again at 4000× *g* for 10 min at 4 °C to remove remaining cells. Sera were stored at −80 °C until use.

#### 2.2.2. Isolation of PBMCs

Briefly, in a 15 mL Falcon tube, 5 mL of prediluted blood (1:1 in phosphate buffer saline (PBS), pH 7.2) was carefully layered over an equal volume of the density gradient Histopaque^®^ 1077 solution (Sigma Aldrich, Schnelldorf, Germany). Tubes were centrifuged at 400× *g* for 30 min. The mononuclear cells were collected at the interphase layer and washed twice with three volumes of PBS containing 2% heat-inactivated fetal calf serum solution (PBS/2% FCS) [12].

#### 2.2.3. Plasma Separation

Two milliliters of heparinized blood were centrifuged at 2000× *g* for 15 min to remove cells and platelets. The resulting supernatant (plasma) was divided into 0.5 mL aliquots and stored at −20 °C until use.

### 2.3. RNA Extraction from Plasma and PBMCs

Total RNA was extracted from plasma and PBMCs using the GeneJET Viral DNA/RNA purification kit (Catalog No. K0821, ThermoFisher Scientific, Waltham, MA, USA) according to the manufacturer’s instructions [13].

### 2.4. Detection of HCV RNA in Plasma and PBMCs

The Bosphore^®^ HCV Quantification kit V3 (Catalog No. ABHCQ3 (100 rxn/box), Anatolia Geneworks, Istanbul, Turkey) was used to detect hepatitis C virus RNA in human plasma and PBMCs, encompassing all HCV genotypes. A region within the 5′UTR was amplified, and fluorescence detection was accomplished using the FAM filter. An internal control was integrated to check PCR inhibition and extraction. The amplification data of the internal control were detected with the HEX filter. The PCR reactions were carried out in a 20 µL final volume, including 12 µL of PCR Master Mix and 8 µL of RNA (sample/standard/positive or negative control). Cycling conditions were 50 °C for 30 min for reverse transcription and 95 °C for 15 min for inactivation of the reverse transcriptase, followed by 50 cycles of 97 °C for 30 s denaturation, 55 °C for 80 s annealing, and 72 °C for 15 s extension. All real-time PCR reactions were performed in a StepOnePlus™ Real-Time PCR thermal cycling instrument (Applied Biosystems, Bedford, MA, USA). The four external quantitation standards were added into the PCR reaction together with the samples and the negative control (PCR-grade water) [14].

### 2.5. Biochemical Measurements

Liver enzymes ALT and AST were measured by enzymatic colorimetric methods [15] using Biodiagnostic kits, Egypt, Catalog No. AL 10 31 (45) and Catalog No. AS 10 61 (45), respectively.

Total and direct bilirubin were measured using Biodiagnostic kits, Egypt, Catalog No. BR 1111 and Catalog No. BR 1112, respectively [16]. Total protein was determined using Biodiagnostic kits, Egypt, Catalog No. TP 20 20 [17], and albumin was determined using Biodiagnostic kits, Egypt, Catalog No. AB 10 10 [18].

### 2.6. Statistical Analysis

Data analysis was performed using the software SPSS (Statistical Package for the Social Sciences) version 20. Quantitative variables were described as their means ± standard deviations and median (range) for nonparametric data. Categorical variables were described as their absolute frequencies and were compared using chi-square and Fisher exact test when appropriate. Kolmogorov–Smirnov (distribution type) and Levene (homogeneity of variances) tests were used to verify assumptions for use in parametric tests. To compare data between two groups, the Mann–Whitney test (for non-normally distributed data) and the independent-sample *t*-test (for normally distributed data) were used. The level of statistical significance was set at *p* < 0.05. A highly significant difference was considered at *p* ≤ 0.001.

## 3. Results

### 3.1. Demographic and Biochemical Parameters of the Studied Groups

The demographic data and biochemical measurements of the case group and their age- and sex-matched controls are summarized in Table 1. There were no statistically significant differences between the 2 groups regarding age (*p* = 0.474) and gender (*p* = 0.478). Total protein was significantly lower in the case group compared to the control group at *p* < 0.001. No significant difference was observed between both groups in serum albumin, ALT, AST, total bilirubin, and direct bilirubin.

### 3.2. The Prevalence of OCI in the Studied Groups

**Figure 1:**
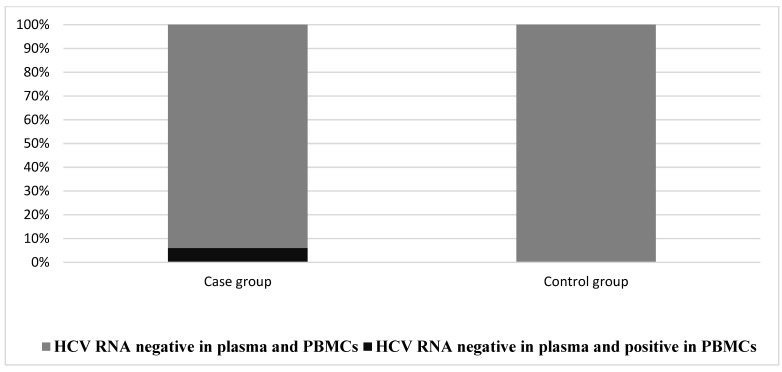
Presence of occult hepatitis C in the case and control groups. Differences were assessed via chi-square test. There was a significantly high level of occult hepatitis C in the case group (6%) compared to the control group (0%) (*p* = 0.029).

According to the occult hepatitis C definition, RNA extractedn from plasma and PBMCs revealed that six out of 100 patients were positive for OCI (HCV RNA-positive from their extracted PBMCs only, without detectable HCV RNA in their plasma), while no cases were found in the control group (*p* = 0.029, Figure 1).

### 3.3. Demographic and Biochemical Measurements in Non-Hodgkin’s and Hodgkin’s Lymphoma in the Case Group

**Figure 2:**
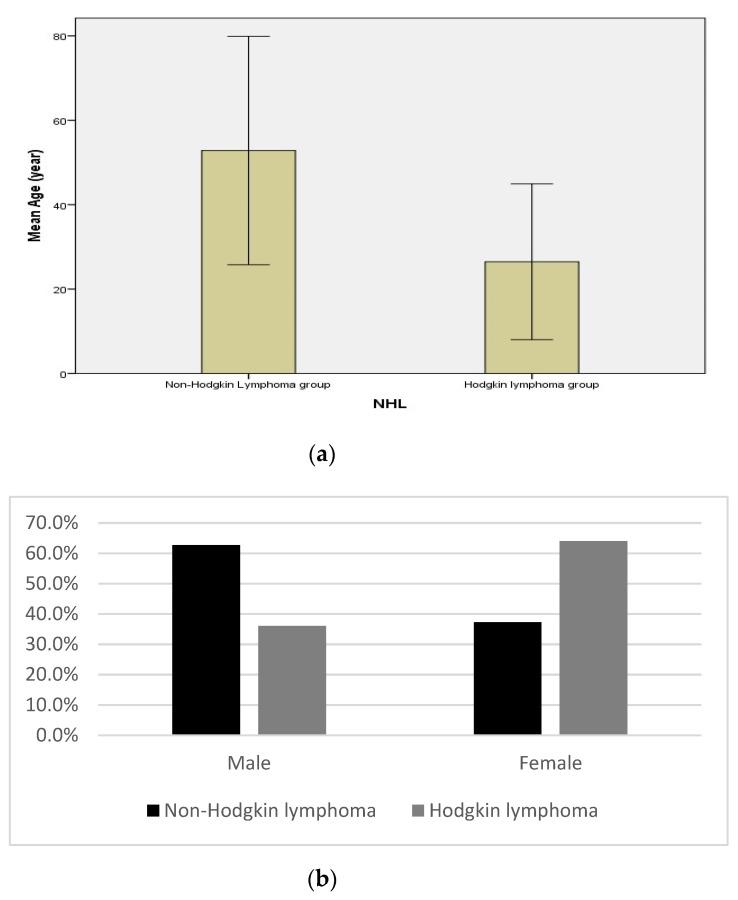
(**a**,**b**) Relationship between the type of lymphoma and demographic data (age and gender) of the studied patients. There was a highly significant difference between patients with non-Hodgkin’s lymphoma with an age mean ± SD of 53.82 ± 13.53 and those with Hodgkin’s lymphoma with an age mean ± SD of 26.48 ± 9.23 (*p* < 0.001), showing that non-Hodgkin’s lymphoma patients were older than Hodgkin’s lymphoma patients, as described in (**a**). For gender, we found a significant difference between the non-Hodgkin’s lymphoma group representing 62.7% males compared to the Hodgkin’s lymphoma group representing 36% males (*p* = 0.02), as shown in (**b**).

In describing the relationship between the type of lymphoma and demographic data (age and gender) of the studied patients, we observed a significant difference in age between the two types of lymphoma (patients with NHL were older than patients with HL, 53.82 ± 13.53 vs. 26.48 ± 9.23, respectively, at *p* = 0.001, Figure 2a). Furthermore, there was a significant difference in gender between the NHL and HL groups at *p* = 0.02. Males represented 62.7% of NHL patients versus 36% within the HL group (Figure 2b).

Additionally, the non-Hodgkin’s lymphoma group had lower serum albumin (*p* = 0.006) and higher serum AST (*p* = 0.031) compared to the Hodgkin’s lymphoma group. The two groups showed no significant differences in total protein, ALT, and total and direct bilirubin (Table 2).

### 3.4. The Prevalence of OCI in Patients with Different Types of Lymphoma

There was no significant difference between NHL and HL patients regarding presence of occult hepatitis C (six patients (8%) within NHL had occult C, while none within HL had occult C) (Table 3).

### 3.5. Demographic and Biochemical Parameters in the Case Group According to the Presence or Absence of OCI

There were no significant differences regarding age and gender between patients with and without occult hepatitis C in the case group (Table 4). However, patients with OCI had lower total protein (*p* = 0.007) and albumin (*p* = 0.03) and a higher serum ALT (*p* = 0.021) and AST (*p* = 0.006) compared to patients without OCI (Table 4). No significant difference was observed between the two groups in levels of total and direct bilirubin.

## 4. Discussion

Occult HCV infection is a new type of chronic HCV infection [19]. OCI was associated with the presence of HCV RNA in peripheral blood mononuclear cells and liver cells but no HCV RNA in serum. OCI can be detected in anti-HCV positive patients with normal serum liver enzyme levels and in anti-HCV-negative patients with persistently elevated liver enzymes [20].

The gold-standard assay for the diagnosis of OCI is a liver biopsy, but this invasive approach has several hazards [21]. OCI detection in PBMCs is a noninvasive technique that successfully diagnoses 60% of OCI cases [22].

The link between OCI and lymphoproliferative disorders has been proven, implying a role in lymphomagenesis [10].

In 2017, Cuellar et al., proved that occult infection adhered to the same HCV-induced lymphomagenesis theory, which included (a) continuous external stimulation of lymphocyte receptors by viral antigens and subsequent proliferation, (b) HCV replication in B cells with an oncogenic effect mediated by intracellular viral proteins, and (c) the “hit and run” theory or permanent B-cell damage caused by a transiently intracellular virus [23].

A higher expression of low-density lipoprotein receptors plays a critical role in the entry of HCV into PBMCs, as well as replication of the virus in lymphocytes and monocytes with HCV persistence in PBMCs [24].

In the current study, there was a significant decrease in total protein levels in the case group when compared to the control group. This result can be explained by the fact that recurrent viral infection is the most common cause of hypoproteinemia. Our results are in agreement with the study performed by Planinc-Peraica et al., who reported low levels of total protein fractions (alpha 1, alpha 2, and beta globulin) in lymphoma patients [25].

A previous study reported the presence of OCI in apparently healthy subjects, where 20% of NHL patients had OCI and 4% of the control patients were positive for OCI [10]. The presence of OCI in apparently healthy individuals is alarming, as the infection can spread. In this study, we compared the prevalence of OCI in lymphoma patients and healthy subjects. OCI was present in 6% of the lymphoma patients and none of the healthy subjects. These findings may reflect the significant effort of the National Committee for the Control of Viral Hepatitis (NCCVH), which developed a national strategy to develop effective direct-acting antiviral agents paid for by the Egyptian government in 2014, with successful cure rates exceeding 90% as reported in 2018 [26].

Our results revealed that all positive occult hepatitis C patients were non-Hodgkin’s lymphoma type. This agrees with De Re et al., who described that HCV-associated NHLs evolved from germinal center (GC) or post-GC B cells that acquired somatic hyper mutation in response to a common antigen, as evidenced by their limited immunoglobulin gene repertoire [27]. Furthermore, Defrancesco et al. reported that HCV-E2 glycoprotein, which strongly corresponds with viremia and antibody responses against HCV, is one of the most critical antigens implicated in chronic B-cell activation [28]. Lastly, the ability of HCV to cause high mutation frequency supports its causative role in tumor formation via a “hit-and-run” mechanism [29].

OCI may predispose to reactivation of clinically evident HCV infection, especially where the immune system is compromised due to comorbidities or suppressive treatment [24].

In this study, patients with OCI had significantly lower levels of total protein and serum albumin. Furthermore, ALT and AST levels were significantly higher in patients with OCI compared to patients without OCI. Our results agree with those of Saad et al., who suggested that OCI can induce mild liver disease. For that reason, follow-up is recommended for OCI patients to monitor the progression to overt disease [30].

Lastly, in LPD patients, repeated blood transfusions are a major risk factor for the development of HCV and OCI. In this case, there are two accepted probabilities; HCV and OCI can be acquired through repeated infected blood transfusions in LPD patients [31] or LPD can develop as a disorder secondary to HCV infection [10]. It is possible that the treatment of OCI patients using antiviral therapies can improve the clinical condition of LPD patients.

## 5. Conclusions

In conclusion, the current study showed that six out of 100 patients with lymphoma disorders had occult HCV infection. Because all OCI-confirmed lymphoma patients were NHL-type and treatment implications are limited following detection of OCI, HCV screening, preferably with HCV RNA, is important in all NHL patients for its treatment implications. Future studies on a larger number of patients are recommended to assess the impact of OCI on lymphoma progression while also focusing on the potential of OCI to accelerate liver disease progression.

## Figures and Tables

**Table 1:** Demographic and biochemical parameters of the studied groups.

Parameter	Groups		*p-*Value
	Case Group	Control Group	
	*N* = 100 (%)	*N* = 100 (%)	
Age (year):			
Mean ± SD	46.24 ± 17.0	44.72 ± 12.65	0.474
Gender:			
Male	56 (56%)	51 (51%)	0.478
Female	44 (44%)	49 (49%)	
T. protein (g/dL)			
Mean ± SD	7.17 ± 0.49	7.46 ± 0.45	<0.001 **
S. albumin (g/dL)			
Mean ± SD	4.26 ± 0.52	4.31 ± 0.4	0.476
ALT:			
Median	22	21.5	0.487
Range	10–90.5	14–27	
AST:			
Median	24	23.5	0.247
Range	12.2–100.2	15–33	
T. bilirubin:			
Median	0.6	0.6	0.11
Range	0.21–2.19	0.21–0.65	
D. bilirubin:			
Median	0.17	0.15	0.164
Range	0.08–0.63	0.14–0.4	

T. protein: total protein, S. albumin: serum albumin, ALT: alanine aminotransferase, AST: aspartate aminotransferase, T. bilirubin: total bilirubin, D. bilirubin: direct bilirubin. ** *p* ≤ 0.001, statistically highly significant.

**Table 2:** Biochemical parameters of the two types of lymphoma in the case group.

Parameter	Lymphoma		*p*-Value
	Non-Hodgkin’s	Hodgkin’s	
	*N* = 75	*N* = 25	
T. protein (g/dL)			
Mean ± SD	7.13 ± 0.53	7.28 ± 0.34	0.195
S. albumin (g/dL)			
Mean ± SD	4.2 ± 0.55	4.46 ± 0.33	0.006 *
ALT:			
Median	22	20	0.695
Range	10–90.5	10–47	
AST:			
Median	24	22	0.031 *
Range	1–100.2	12.2–38	
T. bilirubin:			
Median	0.65	0.45	0.221
Range	0.21–1.39	0.29–2.19	
D. bilirubin:			
Median	0.17	0.16	0.814
Range	0.08–0.63	0.10–0.26	

T. protein: total protein, S. albumin: serum albumin, ALT: alanine aminotransferase, AST: aspartate aminotransferase, T. bilirubin: total bilirubin, D. bilirubin: direct bilirubin. * *p* < 0.05, statistically highly significant.

**Table 3:** Prevalence of OCI in patients with different types of lymphoma.

Occult C	Lymphoma		*p-*Value
	Non-Hodgkin’s	Hodgkin’s	
	*N* = 75 (%)	*N* = 25 (%)	
Positive	6 (8%)	0 (0%)	
Negative	69 (92%)	25 (100%)	0.322

**Table 4:** Demographic and biochemical parameters in the case group according to the presence or absence of OCI.

Parameter	Occult C		*p-*Value
	Absent	Present	
	*N* = 94 (%)	*N* = 6 (%)	
Age (year):			
Mean ± SD	46.89 ± 17.11	36.0 ± 12.03	0.129
Gender:			
Male	42 (60.9%)	5 (83.3%)	0.401
Female	27 (39.1%)	1 (16.7%)	
T. protein (g/dL)			
Mean ± SD	7.2 ± 0.47	6.65 ± 0.61	0.007 *
S. albumin (g/dL)			
Mean ± SD	4.29 ± 0.5	3.82 ± 0.68	0.03 *
ALT:			0.021 *
Median	21.5	42.5	
Range	10–47	15.4–90.5	
AST:			
Median	23	26.2	0.006 *
Range	12.2–38	23–100	
T. bilirubin:			
Median	0.6	0.6	0.977
Range	0.21–2.19	0.22–1.39	
D. bilirubin:			
Median	0.17	0.17	0.556
Range	0.08–0.63	0.13–0.63	

T. protein: total protein, S. albumin: serum albumin, ALT: alanine aminotransferase, AST: aspartate aminotransferase, T. bilirubin: total bilirubin, D. bilirubin: direct bilirubin. * *p* < 0.05, statistically significant.

## Data Availability

Not applicable.
